# Inflammation and nerve injury minimally affect mouse voluntary behaviors proposed as indicators of pain

**DOI:** 10.1016/j.ynpai.2017.09.001

**Published:** 2017-09-08

**Authors:** Tayler D. Sheahan, Edward R. Siuda, Michael R. Bruchas, Andrew J. Shepherd, Durga P. Mohapatra, Robert W. Gereau, Judith P. Golden

**Affiliations:** a Washington University Pain Center and Department of Anesthesiology, Washington University School of Medicine, St. Louis, MO, United States; b Washington University Program in Neuroscience, Washington University School of Medicine, St. Louis, MO, United States

**Keywords:** Non-reflexive, Stimulus-independent, Wheel running, Gait, Social interaction, Anxiety

## Abstract

It has been suggested that the lack of rodent behavioral assays that represent the complexities of human pain contributes to the poor translational record of basic pain research findings. Clinically, chronic pain interferes with patient mobility and physical/social activities, and increases anxiety symptoms, in turn negatively impacting quality of life. To determine whether these behaviors are similarly influenced by putative pain manipulations in rodents, we systematically evaluated wheel running, locomotion, gait, social interaction, and anxiety-like behavior in models of inflammation and nerve injury in adult C57BL6/J male mice. We demonstrate that inflammation and nerve injury differentially affect voluntary behaviors while mice are hypersensitive to mechanical stimuli. Bilateral Complete Freund’s Adjuvant (CFA)-induced inflammation transiently suppressed wheel running and locomotion and also induced gait deficits. In contrast, spared nerve injury (SNI) altered gait and impaired gross motor coordination. SNI-induced gait changes were not reversed by the analgesic PD123319, an angiotensin II type 2 receptor antagonist, and are therefore likely to be motor-related rather than pain-related. Neither CFA nor SNI significantly altered social interaction or elicited general anxiety-like behavior. Our findings suggest that in contrast to humans, mobility and physical/social activities are minimally altered, if at all, in mice following inflammation or nerve injury.

## Highlights

• Inflammation suppressed wheel running and locomotion, and impaired gait in mice.
• Nerve injury gave rise to gait deficits that are likely motor-, not pain-related.
• Changes in wheel running or gait were unrelated to the degree of hypersensitivity.
• Neither inflammation nor nerve injury altered social interactions or anxiety-like behavior.

## Introduction

Chronic pain is an immense clinical and societal burden with largely unsatisfactory pharmacological treatment options (Gereau et al., 2014, Institute of Medicine, 2011, O’Connor, 2009). Considering the rising opioid epidemic, it is pressing that we develop new, efficacious drug therapies. However, over the past 50 years, the development of novel analgesics has been hindered by the high failure rate of clinical trials (Kissin, 2010, Moore et al., 2013). The lack of rodent pain assays that encompass the complexities of human chronic pain is thought to contribute to the poor translational record of preclinical analgesics (Barrot, 2012, Clark, 2016, Cobos and Portillo-salido, 2013, Mao, 2012, Mogil, 2009, Mogil and Crager, 2004, Rice et al., 2009, Sapunar and Puljak, 2009, Tappe-Theodor and Kuner, 2014, Vierck et al., 2008). Clinically, chronic pain is characterized by sensory, affective, and emotional changes that negatively impact quality of life (McNamee and Mendolia, 2014). To this end, outcomes of interest in clinical trials for pain relief are primarily improved health-related quality of life and functionality, rather than nociceptive thresholds (O’Connor, 2009, Sullivan and Ballantyne, 2016). However, preclinical pain research has historically relied on mechanical and thermal hypersensitivity as a primary outcome in rodent pain models, which represents only one component of human chronic pain. To address this translational gap, substantial efforts have been directed towards the assessment of voluntary behaviors and quality of life measures in rodent pain models that better reflect how pain impacts the lives of patients (Barrot, 2012, Mogil, 2009, Tappe-Theodor and Kuner, 2014).

Clinical studies demonstrate that chronic pain reduces quality of life in part by impairing mobility as well as physical and social activities, and increasing anxiety symptoms (Dysvik et al., 2004, Gureje, 2008, He et al., 2017, Jensen et al., 2007, Nicholson and Verma, 2004, Smith and Torrance, 2012, Wallace et al., 2014). Accordingly, one goal of preclinical pain researchers is to develop and utilize measures of pain-related suppressed and evoked behaviors (Negus et al., 2006). However, preclinical studies evaluating the effects of inflammation and nerve injury on rodent gait, locomotion, social interaction, and anxiety-like behavior have yielded conflicting results. In some cases, inflammation and nerve injury have been shown to alter gait (Chiang et al., 2014, Coulthard et al., 2002, Mogil et al., 2010, Piesla et al., 2009, Pitzer et al., 2016, Pitzer et al., 2016), suppress general locomotion and voluntary wheel running (Cobos et al., 2012, Grace et al., 2014, Kandasamy et al., 2016, Miller et al., 2011, Pitzer et al., 2016, Pitzer et al., 2016, Stevenson et al., 2009, Stevenson et al., 2011), reduce social interactions (Parent et al., 2012, Ren et al., 2015), and/or induce anxiety-like behavior (Dimitrov et al., 2014, Liu et al., 2015, Narita et al., 2006, Refsgaard et al., 2016, Roeska et al., 2009, Yalcin et al., 2011); however, in other cases, these behaviors were unchanged by persistent pain (Cho et al., 2013, Grace et al., 2016, Monassi et al., 2003, Sheahan et al., 2015, Urban et al., 2011). These conflicting results may be the product of differences in study design with respect to species, injury model, behavioral paradigms, etc. Thus, whether these endpoints are valid measures of rodent pain-related behavior remains unresolved.

In the present study, we systematically evaluated voluntary wheel running, locomotion, gait, social interaction, and anxiety-like behavior in two commonly used mouse models of persistent pain: Complete Freund’s Adjuvant-induced inflammation and the spared nerve injury model of neuropathic pain. When appropriate, we tested for correlations between changes in voluntary behavior and mechanical hypersensitivity, a widely used stimulus-evoked/reflexive endpoint. Further, to determine whether changes in voluntary behaviors were pain-related, we tested if they could be reversed with an analgesic. We utilized the angiotensin II type 2 receptor antagonist PD123319, a promising candidate analgesic that has been shown to be effective in a variety of rodent peripheral nerve injury models (Muralidharan et al., 2014, Shepherd et al., 2017, Smith et al., 2014, Smith and Muralidharan, 2015), and is related to the compound EMA401 that has shown efficacy in a phase II clinical trial for neuropathic pain (Rice et al., 2014).

## Materials and methods

### Experimental animals

Animals were cared for in compliance with the National Institutes of Health guidelines and approved by the Animal Studies Committee of Washington University in St. Louis (Protocol Numbers 20130147, 20160097). Experiments were predominantly conducted on adult C57BL/6J male mice bred in house using breeding pairs from Jackson Labs (Bar Harbor, Maine). One cohort of adult experimental animals was obtained directly from Jackson Labs and allowed to acclimate to our animal housing facility for at least 1 week before initiating behavioral testing. Mice were housed with up to 4 cagemates in the animal facility under a 12-h light/dark cycle (6 AM–6 PM) and provided food and water *ad libitum*. Cages were lined with corncob bedding. Behavioral experiments began when mice were 7–9 weeks of age. Throughout experiments, animals were regularly monitored for general health and weighed weekly. At the conclusion of each study, mice were euthanized using a rodent ketamine euthanasia cocktail.

### Experimental models of pain

Throughout all surgical procedures, mice were anesthetized with 2% isofluorane.

Intraplantar Complete Freund’s Adjuvant (CFA) was used as a model of persistent inflammatory pain. Mice received a single bilateral intraplantar hindpaw injection of 20 μL undiluted CFA (1 mg/mL; Sigma, St. Louis, MO). Control mice were similarly injected bilaterally with 20 μL of 0.9% sterile saline. Behavioral testing began as soon as 4 h post injection.

Unilateral spared nerve injury (SNI) was performed as described previously (Decosterd and Woolf, 2000, Richner et al., 2011, Sheahan et al., 2015). Akron lidocaine hydrochloride jelly (2%; Vessel Medical, Greenville, SC) was applied topically to the incision site. The three branches of the sciatic nerve were exposed by separating the biceps femoris muscle, and the common peroneal and tibial branches were ligated with silk suture and cut distal to the ligation, taking care not to manipulate the sural nerve. Sham operation comprised a skin incision over the biceps femoris muscle. For all operations, the skin incision was closed with staples that were removed on postoperative day (POD) 7, once wounds had healed. Allowing time for post-operative recovery, behavioral testing began no sooner than POD 4. Upon completion of behavioral studies, SNI mice were dissected to confirm that the sural nerve was not included in or damaged by the ligation surgery. If the sural nerve was not intact, animals were excluded from the study (4% of SNI mice).

### CFA-induced paw edema

Paw thickness (in millimeters) was measured using 150 mm stainless dial calipers (Chicago Brand, Medford, OR). Paw thickness was measured at baseline, as well as post-intraplantar injection of either CFA or saline.

### Behavioral studies

For each experimental cohort, animal groups were randomized and the experimenter was blind to injury and/or drug treatment groups until the completion of data analysis. Unless otherwise specified, prior to behavioral testing, mice were acclimated to the testing apparatus (von Frey) or the testing room within their homecages (voluntary wheel running, open field, rotarod) for 1–2 h with low-level white noise. In cases where both baseline and post-injury measurements were made, data for each mouse is reported as normalized to baseline (von Frey, voluntary wheel running, gait analysis). Experiments were performed during the light cycle between 8 AM and 5 PM.

Sample sizes for each behavioral endpoint were modeled off of group sizes required to observe physiologically significant behavioral effects in previously published rodent studies of voluntary behaviors (Pitzer et al., 2016, Pitzer et al., 2016, Siuda et al., 2016). Where correlation analyses were planned, additional animals were tested to meet the adequate sample size to make comparisons between behavioral endpoints. Cohorts were comprised of 10–15 mice from a single source (either in house or Jackson Labs). At least two cohorts of animals were tested for each behavioral endpoint to obtain the appropriate sample size and evaluate whether injury- or drug-induced changes produced similar effects across cohorts. All CFA cohorts were bred in house and all but one SNI cohort (evaluated in von Frey, voluntary wheel running, and the social interaction assay) was bred in house. Similar SNI-induced behavioral effects were observed across cohorts regardless of the animal source.

#### Mechanical sensitivity (von Frey)

Mechanical sensitivity was measured using the up-down method of the von Frey test (Chaplan et al., 1994). Prior to baseline behavioral testing, mice were habituated to the elevated mesh grid in Plexiglass boxes for 2 h/day for 2 days. On testing days, calibrated filaments (North Coast Medical Inc., Gilroy, CA) were applied to the plantar surface of the hindpaw. In SNI studies, baseline and post-operative mechanical sensitivity was measured on the lateral aspect of the hindpaw (Duraku et al., 2012). In all studies, 3 trials were conducted on each paw, with at least 5 min between testing opposite paws, and at least 10 min between testing the same paw. Mechanical withdrawal thresholds of each paw were calculated by averaging values obtained across trials. We established a von Frey threshold inclusion criterion to ensure that an adequate dynamic range existed for detecting mechanical hypersensitivity following injury. In order for an animal to be tested post-injury, baseline thresholds had to be greater than or equal to 0.20 g. Of the total number of mice that underwent baseline testing, 98% of CFA mice (tested on the center of the paw) and 78% of SNI mice (tested on the lateral aspect of the paw) met the inclusion criterion.

#### Voluntary wheel running

Voluntary wheel running was quantified using wireless low-profile running wheels (Med Associates, Fairfax, VT). To reduce environmental novelty, mice were first acclimated to individual cages with locked running wheels for 2 h/day for 2 days. Running wheels were then unlocked for baseline behavioral testing. Distance travelled was recorded for 2 h without the experimenter in the room. Mice were classified as non-runners if they ran less than 200 m during the baseline session and were not tested for voluntary wheel running post-injury. Of all animals that underwent baseline testing, 10% were non-runners. Wheel running experiments were conducted during the light cycle between 11 AM and 1 PM.

#### Social interaction

Social interaction was measured using a social approach assay described previously (Siuda et al., 2016). Room lighting was ∼200 lux. The black, plastic testing arena (52.5 L × 25.5 W × 25.5 H cm), the bottom of which was covered with corncob bedding, consisted of two-chambers, each containing an inverted metal, mesh pencil cup. One pencil cup was arbitrarily designated to lie within the social zone. The social zone was defined as a circle equal to twice the diameter of the pencil cup. Prior to testing, mice were acclimated to a silent room in their homecages for 1 h. In the baseline trial, the test mouse moved freely within the arena for 10 min once the experimenter left the room. EthoVision XT video tracking software (Noldus, Cincinnati, OH) was used to monitor animal movement and quantify time spent within the social zone. Following the baseline trial, the test mouse was returned to its homecage for 30 min. An unfamiliar, age-, sex-, and strain-matched conspecific stimulus mouse was then placed beneath the social zone pencil cup. In the social trial, the test animal was reintroduced into the arena for 10 min and the time spent in the social zone was recorded. Test mice could see, hear, and smell, but not physically interact with the stimulus mouse within the pencil cup. Social interaction scores were determined by dividing the amount of time the test mouse spent in the social zone during the social trial by the amount of time the test mouse spent in the social zone during the baseline trial.

#### Locomotor activity and anxiety-like behavior (open field)

Locomotor activity was quantified in an open field chamber equipped with a Versamax Animal Activity Monitoring System (AccuScan Instruments Inc., Columbus, OH) under normal laboratory light (∼770 lux). Following acclimation, mice were individually placed into the center of the open field and allowed to explore the chamber for 1 h once the experimenter left the room. Total distance traveled and time spent moving were calculated for the 42 L × 42 W × 20 H cm chamber. Anxiety-like behavior was evaluated by quantifying the percentage of time spent in the center zone of the open field arena over the 1 h testing period. The center zone was defined as a square comprising 40% of the arena. As open field activity is largely driven by exploratory behavior of a novel environment (Cummins and Walsh, 1976), mice were only tested in the open field once. Thus, open field activity across multiple post-CFA timepoints was acquired from separate cohorts of mice.

#### Gross motor function (rotarod)

Motor coordination and balance was determined using an accelerating rotarod (Ugo Basile, Varese, Italy). First, a training session involving 120 s on a non-accelerating rotarod was conducted, followed by 1 h of rest with mice in their homecages. Then 5 consecutive trials with 5 min rest intervals between trials were performed. Latency to fall as the rotarod accelerated from 4 to 40 rpm in 30 s increments over 5 min was recorded.

#### Catwalk automated gait analysis

Gait analysis was performed using the Catwalk XT 10.5 system (Noldus, Cincinnati, OH). Briefly, the Catwalk XT system consists of an elevated, enclosed glass platform (130 L × 7 W cm) and a high-speed camera (GEViCAM GP-2360C). Green light is internally reflected into the glass platform and light is emitted downward only when pressure is placed upon the glass (i.e. by an animal’s paw). The high-speed camera detects emitted light intensity per pixel, and the accompanying Catwalk XT software acquires and analyzes gait parameters. Gait analysis data were acquired using the following experimental settings: camera gain: 13–15 dB, green light intensity threshold: 0.12, run duration: 0.5–5.0 s, run maximum variation: 60%. In some cases, the ipsilateral hindpaw of SNI mice was not detectable using these experimental settings and these animals were ineligible for analysis.

The day prior to baseline behavioral testing, mice were acclimated to the Catwalk platform over two 15 min sessions separated by 30 min. On testing days, mice were habituated to the testing room for at least 30 min and behavioral testing was conducted in a completely dark, silent room. Mice voluntarily traversed the enclosed glass platform. At each testing timepoint, 4 compliant runs were obtained and averaged per mouse. Postoperative Catwalk testing on SNI animals began after surgical staples were removed on POD 7, as the staples could have subtle effects on gait.

Parameters obtained and reported from Catwalk gait analysis include:

*Paw pressure (max contact, mean intensity)*: Average print intensity (a.u.) when the paw is making maximum contact with the glass.
*Run speed*: Average body speed (cm/s)
*Stance phase*: Duration (s) that paw is in contact with the glass platform
*Swing phase*: Duration (s) that paw is *not* in contact with the glass platform
*Step duration*: Sum (s) of stance and swing phase
*Fraction stance phase*: Stance phase divided by step duration
*Fraction swing phase*: Swing phase divided by step duration
*Maximum contact area*: Maximum area (cm^2^) of the paw that contacts the glass platform

### Drugs and drug administration

PD123319 ditrifluoroacetate (Tocris, Minneapolis, MN), also known as EMA200, was dissolved in 0.9% sterile saline and injected at 10 mg/kg, i.p. Vehicle controls were injected with an equal volume of saline. Notably, previous studies demonstrate that systemic administration of 10 mg/kg PD123319 attenuates hypersensitivity without impairing motor coordination in the rotarod test (Pechlivanova et al., 2013, Smith et al., 2014). The effects of PD123319 on mechanical hypersensitivity and Catwalk hindpaw pressure were evaluated over 3 testing sessions per behavioral assay: pre-drug, drug, and post-drug sessions. Each testing session was separated by 24–48 h. All experiments evaluating the effects of PD123319 were performed between POD 7 and POD 41, when SNI mice were known to be hypersensitive. Mechanical hypersensitivity testing sessions were performed on either POD 12–16 or 26–30 and results from these timepoints were pooled together. Catwalk hindpaw pressure testing sessions were performed on POD 9–12. Behavioral testing took place 1.5–2.5 h post injection, the time frame at which peak analgesia with respect to mechanical hypersensitivity has been reported (Shepherd et al., 2017, Smith et al., 2014).

### Statistical analyses

All data were analyzed using Excel (Microsoft, Redmond, WA) and Prism (GraphPad Software, Inc., La Jolla, CA) and are presented as mean ± SEM. Significance was defined as *p* < 0.05 a priori. Post-injury withdrawal thresholds, running distances, Catwalk gait parameters, and paw thickness are normalized to baseline values measured prior to inflammation or nerve injury. Figure legends indicate the group size and statistical test for each experiment. Briefly, data comparing the effect of CFA or SNI on paw thickness, body weight, and behavior over time were analyzed with a Student’s t-Test and Holm-Sidak correction for multiple comparisons, when appropriate. The effects of PD123319 on SNI-induced behavioral changes were analyzed using a two-way repeated-measures (RM) ANOVA, post hoc Student’s t-Tests, and Sidak correction for multiple comparisons. Pearson correlation coefficients were determined to evaluate the relationship between CFA- or SNI-induced mechanical hypersensitivity and changes in voluntary behaviors.

## Results

### CFA induced paw edema and mechanical hypersensitivity

**Fig. 1:**
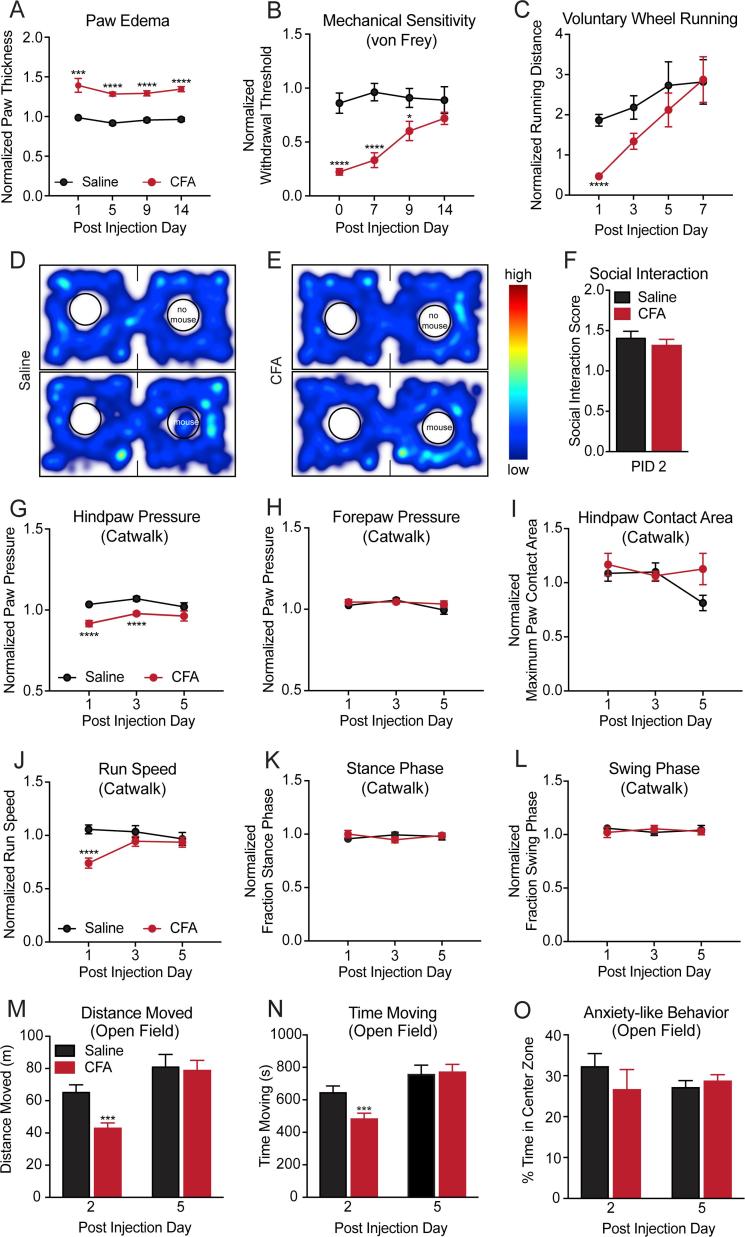
Bilateral CFA suppressed locomotion and altered gait, but did not affect social interaction or anxiety-like behavior. (A) Bilateral intraplantar injection of Complete Freund’s Adjuvant (CFA) produced significant paw edema that persisted for at least 14 days compared to injection of saline (n = 10–17/group). (B) CFA mice displayed a significant reduction in mechanical hindpaw withdrawal thresholds as soon as 4 h post injection (day 0) through post injection day (PID) 9 compared to saline mice (n = 10–17/group). (C) Voluntary running was significantly reduced by CFA on PID 1 compared to saline (n = 11–23/group). Representative heatmaps from the social interaction test of (D) saline and (E) CFA mice in the absence (top) and presence (bottom) of an unfamiliar conspecific. Scale bar indicates total time spent in each arena location. Presence within circles reflects time spent atop the pencil cups. (F) Social interaction scores were equivalent between CFA and saline mice on PID 2 (n = 12/group). (G, H) Catwalk gait analysis demonstrated that CFA significantly decreased hindpaw pressure though PID 3 compared to saline, yet no changes in forepaw pressure were observed (n = 12/group). (I) Despite the paw edema of CFA mice, hindpaw contact area with the Catwalk platform was equivalent between CFA and saline mice. (J, K, L) CFA caused a significant reduction in run speed across the Catwalk platform on PID 1; however no differences in stance phase or swing phase were observed between CFA and saline mice. (M, N) CFA significantly reduced distance moved and time spent moving in an open field compared to saline on PID 2 (n = 11–13). (O) CFA mice did not display anxiety-like behavior in an open field compared to saline mice at either timepoint tested. Data are presented as mean ± SEM. Student’s t-Test, Holm-Sidak correction for multiple comparisons, when appropriate. ^*^p < 0.05, ^**^p < 0.01, ^***^p < 0.001, ^****^p < 0.0001, as compared with saline mice.

To determine the effects of persistent inflammation on rodent stimulus-evoked and voluntary behaviors, we utilized the intraplantar Complete Freund’s Adjuvant (CFA) model of inflammatory pain (Iadarola et al., 1988, Ren and Dubner, 1999). Following bilateral intraplantar injections of CFA, we observed significant thickening of the hindpaws that persisted for at least 14 days relative to saline-injected controls (Fig. 1A). Intraplantar CFA is known to produce robust hypersensitivity that persists for at least 1–2 weeks (Cobos et al., 2012, Iadarola et al., 1988, Ren and Dubner, 1999, Smith et al., 2016). Similarly, we observed a significant reduction in hindpaw mechanical withdrawal thresholds in CFA mice compared to saline mice (Fig. 1B). Mechanical hypersensitivity was detectable on post injection day (PID) 0 (∼4 h after the injection) through PID 9. Interestingly, CFA-induced mechanical hypersensitivity recovered by PID 14 despite persistent paw edema.

### CFA suppressed voluntary wheel running

To determine whether persistent inflammation alters voluntary behaviors, we tested mice between PID 0 and 7, when CFA-induced mechanical hypersensitivity was most robust. We first assessed whether inflammation suppressed voluntary wheel running. In order to decrease the likelihood of exercise-induced analgesia (Cobianchi et al., 2017, Koltyn, 2000), wheel running distances were quantified every other day beginning 1 day after injection. We initially tested whether unilateral intraplantar CFA suppressed voluntary wheel running. In alignment with a previous report (Cobos et al., 2012), unilateral CFA did not affect wheel running activity compared to saline (data not shown). We next tested whether bilateral CFA affected voluntary wheel running. Relative to saline, bilateral CFA produced a significant reduction in voluntary wheel running on PID 1 (Fig.1C). Wheel running of CFA mice began to recover on PID 3 and reached that of saline mice by PID 7, despite continued CFA-induced mechanical hypersensitivity through PID 9. The time course of recovery from CFA-induced suppression of voluntary wheel running closely resembles that reported by others (Cobos et al., 2012, Grace et al., 2014, Kandasamy et al., 2016, Pitzer et al., 2016), suggesting that the effects of CFA on voluntary wheel running are consistent and reproducible. As bilateral CFA administration was required to observe suppression of wheel running, all subsequent investigations of the effects of inflammation on voluntary behavior were conducted using bilateral CFA injections.

### CFA did not affect social interactions

To determine whether persistent inflammation decreased social interaction, we used a behavioral model of social approach (Silverman et al., 2010, Siuda et al., 2016). Social interaction scores of CFA mice with an unfamiliar age-, sex-, and strain-matched conspecific were equivalent to saline mice on PID 2 (Fig. 1D-F). Our findings support previous work demonstrating that acutely after injection, CFA does not affect social interactions of mice that are hypersensitive to mechanical stimuli (Liu et al., 2015).

### CFA induced gait alterations

To evaluate whether bilateral CFA elicited static and/or dynamic gait deficits, we utilized the Catwalk XT gait analysis system. With respect to static gait parameters, compared to saline, CFA significantly reduced hindpaw pressure through PID 3 (Fig. 1G) without altering forepaw pressure (Fig. 1H). Paw pressure was determined at the point of maximum contact of the paw with the Catwalk platform. Therefore, we evaluated whether CFA-induced paw edema could be affecting hindpaw pressure by increasing maximum hindpaw contact area. However, compared to saline, CFA administration did not significantly alter hindpaw maximum contact area (Fig. 1I).

With respect to dynamic gait parameters, CFA significantly decreased run speed on PID 1 compared to saline (Fig. 1J). Many gait parameters are dependent on run speed (Batka et al., 2014). Therefore, stance phase and swing phase were calculated as a fraction of the total step duration, the combined duration of the stance and swing phases. No changes in stance phase or swing phase were observed following CFA (Fig. 1K, L). In contrast to these findings, previous reports of rodent gait analysis following unilateral intraplantar CFA demonstrate a broader effect of inflammation on gait. For instance, reduced stance phase and prolonged swing phase of the injected hindlimb have been proposed to reflect pain avoidance behavior (Coulthard et al., 2002, Piesla et al., 2009, Pitzer et al., 2016). In our studies, however, bilateral injury likely precluded the ability to observe possible inflammation-induced avoidance behaviors in hindpaw gait parameters. Collectively, our findings demonstrate that bilateral CFA produces transient, yet significant changes in a subset of static and dynamic gait parameters.

### CFA induced changes in open field behavior

To determine whether persistent inflammation affected locomotor and/or anxiety-like behavior of mice, open field activity was evaluated. On PID 2, CFA mice displayed significantly decreased distance moved and time spent moving within the open field compared to saline mice (Fig. 1M, N). These locomotor deficits were no longer present in mice tested on PID 5. Similarly, Refsgaard et al. report that open field locomotor behavior is unchanged on PID 4 following unilateral intraplantar CFA (Refsgaard et al., 2016). In the open field test, anxiety-like behavior is expressed as a reduction in the proportion of time spent in the center zone of the open field compared to a control condition. In our studies, time spent in the center of the open field was comparable between CFA and saline mice on all testing days (Fig. 1O). These results suggest that mice do not exhibit anxiety-like behavior in the open field after inflammation, and are supported by previous findings (Liu et al., 2015, Urban et al., 2011).

### SNI induced mechanical hypersensitivity

**Fig. 2:**
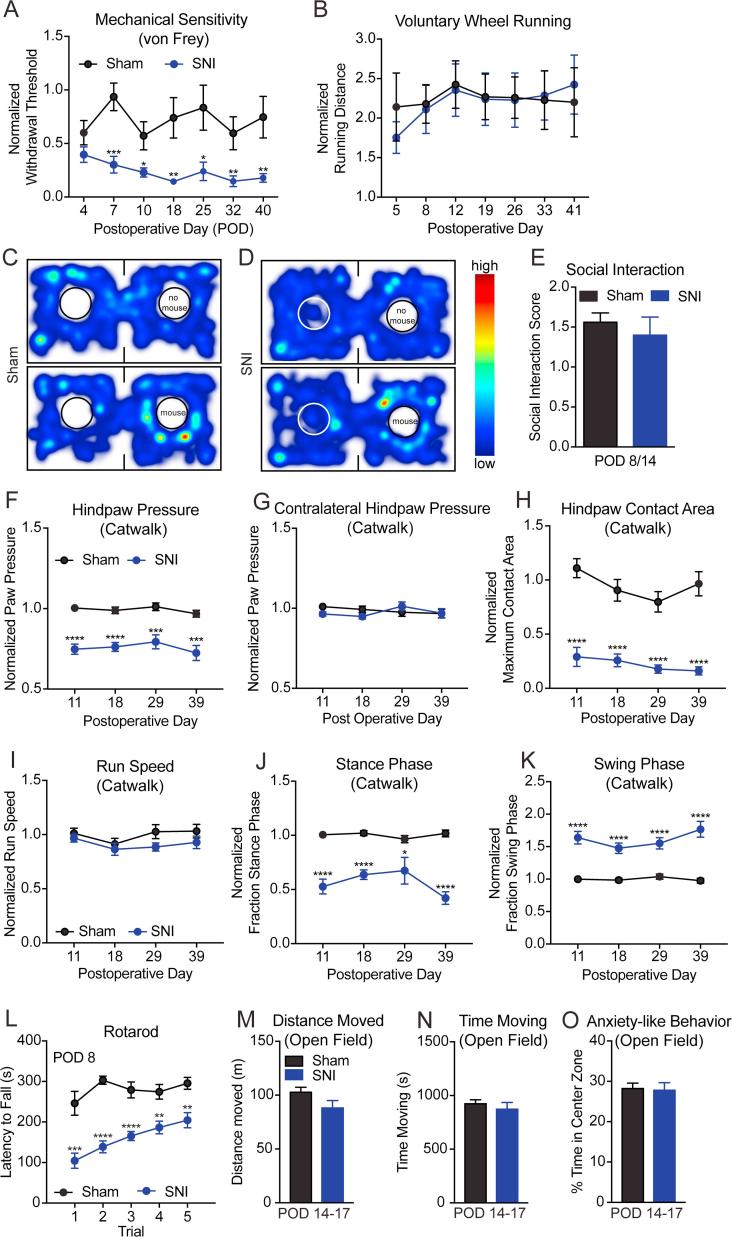
SNI produced gait alterations, but did not influence locomotion, social interaction, or anxiety-like behavior. (A) Spared nerve injury (SNI) caused a significant reduction in hindpaw mechanical withdrawal thresholds compared to sham operation on postoperative days (POD) 7–40 (n = 10–17/group). (B) SNI and sham mice exhibited equivalent wheel running activity at each testing timepoint between POD 5 and POD 41 (n = 12–18/group). Representative heatmaps from the social interaction test of (C) sham and (D) SNI mice in the absence (top) and presence (bottom) of an unfamiliar conspecific. Presence within circles reflects time spent atop the pencil cups. Scale bar indicates total time spent in each arena location. (E) Social interaction scores were equivalent between sham and SNI mice on POD 8/14 (n = 15/group). (F, G) Catwalk gait analysis demonstrated a significant reduction in ipsilateral hindpaw pressure, but no change in contralateral hindpaw pressure of SNI mice relative to sham mice on POD 11 through POD 39 (n = 10–23/group). (H) SNI significantly decreased hindpaw contact area compared to sham surgery. (I) Catwalk run speed did not differ between SNI and sham mice. (J, K) SNI mice had a significantly shorter stance phase and significantly longer swing phase relative to sham mice on POD 11 through POD 39. (L) SNI mice were impaired in the rotarod test, and had a significantly shorter latency to fall compared to sham mice across all trials on POD 8 (n = 10–12/group). (M, N) SNI did not affect distance moved or time spent moving in the open field test on POD 14–17 (n = 12–16). (O) SNI mice did not exhibit anxiety-like behavior in the open field test compared to sham mice on POD 14–17. Data are presented as mean ± SEM. Student’s t-Test, Holm-Sidak correction for multiple comparisons, when appropriate. ^*^p < 0.05, ^**^p < 0.01, ^***^p < 0.001, ^****^p < 0.0001, as compared with sham mice.

We next tested whether nerve injury also caused changes in voluntary behavior. To determine the effects of nerve injury on rodent stimulus-evoked and voluntary behaviors, we utilized the spared nerve injury (SNI) model of neuropathic pain. SNI has been shown to produce prolonged mechanical hypersensitivity in the lateral aspect of the hindpaw (Decosterd and Woolf, 2000, Sheahan et al., 2015, Urban et al., 2011). We similarly observed a significant reduction in hindpaw mechanical withdrawal thresholds of SNI mice compared to sham-operated mice by postoperative day (POD) 7, which persisted through at least POD 40 (Fig. 2A).

### SNI did not suppress voluntary wheel running or social interaction

To test whether nerve injury suppressed voluntary wheel running, running distances were quantified at multiple postoperative timepoints between POD 5 and POD 41. At each timepoint, SNI mice and sham mice displayed equivalent wheel running activity (Fig. 2B). These findings were obtained during the light cycle and are supported by our previous observations that SNI mice ran equivalent distances to uninjured mice when given wheel access for either 2 or 12 h per night, the time at which mice are most active (Sheahan et al., 2015).

Similarly, we tested whether nerve injury suppressed social interaction with an unfamiliar age-, sex-, and strain-matched conspecific. We observed that social interaction scores were unchanged in SNI mice relative to sham mice on POD 8 or 14 (Fig. 2C-E). Interestingly, previous studies show SNI-induced decreases in rodent social interaction at postoperative timepoints more acute (POD 5) (Ren et al., 2015, Zhou et al., 2015), but not long-term (POD 40) (Urban et al., 2011), than those we tested. Taken together, our results demonstrate that despite ongoing mechanical hypersensitivity, neither voluntary wheel running nor social interaction were suppressed by nerve injury.

### SNI induced gait and motor deficits

Using the Catwalk gait analysis system, we tested whether nerve injury altered gait. Significant changes in both static and dynamic gait parameters were observed after SNI from POD 11 to 39. Analysis of static gait parameters revealed that compared to sham-operated mice, ipsilateral hindpaw pressure of SNI mice was significantly decreased (Fig. 2F). However, no changes in contralateral hindpaw pressure were observed (Fig. 2G). As with other rodent sciatic nerve injury models (Rigaud et al., 2008, Ye et al., 2014), SNI gave rise to cupping of the hindpaw, highlighted by a significant reduction in hindpaw maximum contact area compared to sham-operated mice (Fig. 2H). Analysis of dynamic gait parameters demonstrated that the run speed of SNI mice was comparable to sham mice (Fig. 2I). However, SNI mice displayed a significantly shorter stance phase (Fig. 2J), as well as significantly longer swing phase (Fig. 2K) relative to sham controls. Interestingly, in contrast to bilateral CFA, all static and dynamic gait deficits following SNI persisted as long as mechanical hypersensitivity, through at least POD 39.

Multiple studies have demonstrated gait deficits following nerve injury in rodents (Chiang et al., 2014, Mogil et al., 2010, Piesla et al., 2009, Pitzer et al., 2016). However, whether these effects represent a pain-avoidance behavior or simply a motor deficit is uncertain (Tappe-Theodor and Kuner, 2014). To address this, we tested gross motor coordination of nerve-injured mice using the accelerating rotarod test on POD 8. Indeed, SNI mice displayed a significant motor impairment across all test trials. SNI mice had a significantly shorter latency to fall off the rotarod than sham mice (Fig. 2L). Urban et al. also observed SNI-induced motor impairment in the rotarod test (Urban et al., 2011). Thus, SNI-induced gait deficits may be the product of gross motor deficits.

### SNI did not induce changes in open field behavior

To determine whether nerve injury suppressed locomotor behavior or induced anxiety-like behavior, we evaluated open field activity on POD 14–17. SNI mice did not differ from sham mice with respect to distance moved (Fig. 2M) or time spent moving (Fig. 2N) in the open field test. Our findings support those of existing studies demonstrating that gross locomotion of mice is unchanged after SNI (Cho et al., 2013, Urban et al., 2011, Zhou et al., 2015). Lastly, percent time spent in the center zone of the open field was equivalent between SNI mice and sham mice (Fig. 2O), suggesting that at this timepoint, nerve-injured mice were not in a general anxiety-like state, as supported by previous findings (Urban et al., 2011).

### Summary of CFA- and SNI-induced changes in voluntary behavior and animal wellbeing

We demonstrated that inflammation and nerve injury differentially affect voluntary behaviors (Table 1). In addition to causing mechanical hypersensitivity, bilateral intraplantar injection of CFA transiently suppressed voluntary wheel running, decreased locomotor activity, and altered static and dynamic gait parameters. However, neither social interactions nor anxiety-like behavior were affected by CFA (Fig. 1). Interestingly, all inflammation-induced changes in voluntary behaviors resolved by PID 5, whereas inflammation-induced mechanical hypersensitivity persisted through PID 9.

**Table 1:** Inflammation and nerve injury differentially affect voluntary behaviors while mice display mechanical hypersensitivity.

Behavioral Endpoint	CFA, bilateral	SNI, unilateral
Mechanical withdrawal threshold	↓	↓
Wheel running	↓	no Δ
Gait	Δ	Δ
Social interaction	no Δ	no Δ
Open field-locomotion	↓	no Δ
Open field-anxiety	no Δ	no Δ

↓ indicates significantly decreased behavior; Δ indicates significant behavioral changes; no Δ indicates absence of significant behavioral changes; CFA, Complete Freund’s Adjuvant; SNI, spared nerve injury.

In contrast, despite robust and persistent mechanical hypersensitivity, SNI did not suppress voluntary wheel running, social interactions, or locomotor activity. Further, SNI did not induce general anxiety-like behavior in the open field. SNI did produce changes in both static and dynamic gait parameters that, like mechanical hypersensitivity, persisted the entire length of our study, through POD 39 (Fig. 2). However, it is possible that these gait alterations are in part the product of a general motor deficit, as suggested by impaired rotarod performance of SNI mice.

**Fig. 3:**
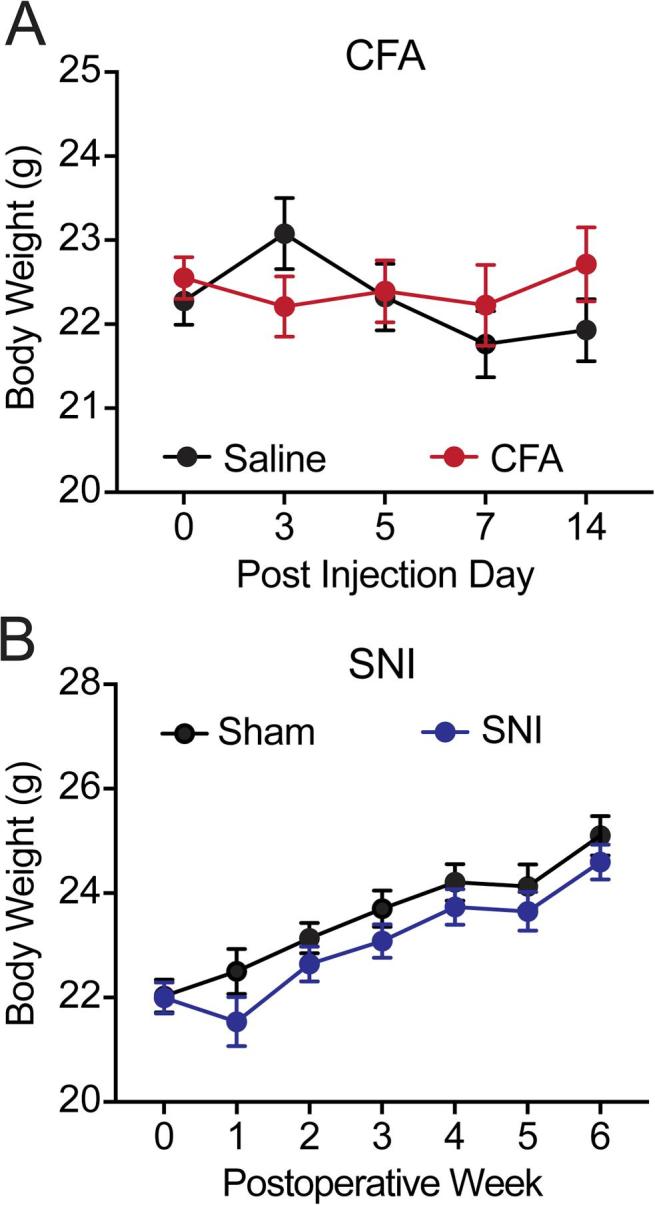
Neither CFA nor SNI affected mouse body weight. (A) Body weight of Complete Freund’s Adjuvant (CFA) mice was equivalent to saline mice from PID 0 through PID 14 (n = 13–42/group). (B) Body weights of spared nerve injury (SNI) and sham mice were equivalent between postoperative weeks 0 and 6 (n = 23–38/group). Data are presented as mean ± SEM. Student’s t-Test, Holm-Sidak correction for multiple comparisons.

In addition to evaluating voluntary behaviors following injury, we also monitored body weight as a measure of general animal health (Burkholder et al., 2012). CFA mice had body weights equivalent to saline mice through PID 14 (Fig. 3A). Likewise, body weights of SNI mice were equivalent to sham mice through postoperative week 6 (Fig. 3B). In summary, despite ongoing mechanical hypersensitivity, inflammation and nerve injury do not negatively impact overall animal health, and have primarily short-lived effects on voluntary behaviors.

### CFA- and SNI-induced changes in voluntary behavior did not correlate with mechanical hypersensitivity

**Fig. 4:**
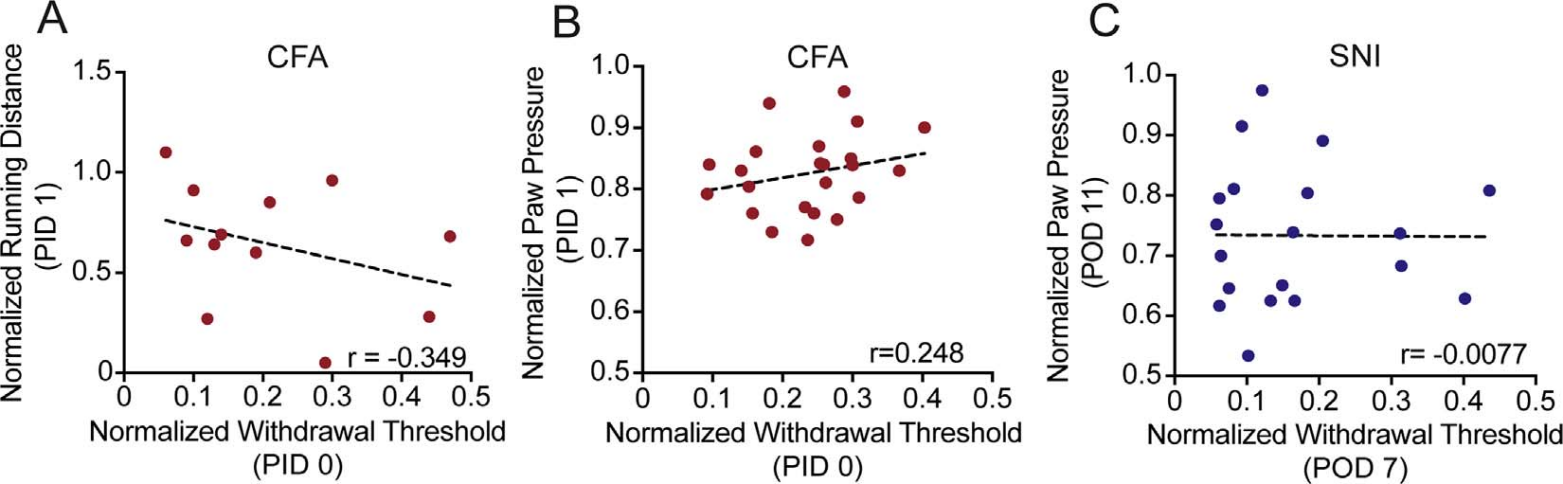
CFA- and SNI-induced changes in voluntary behavior did not correlate with mechanical hypersensitivity. (A) Following bilateral Complete Freund’s Adjuvant (CFA), reductions in hindpaw withdrawal thresholds on post injection day (PID) 0 did not correlate with decreased voluntary wheel running distances (n = 12, r(10) = −0.349, p = 0.267) or (B) reduced Catwalk hindpaw pressure (n = 23, r(21) = 0.248, p = 0.255) measured on PID 1. (C) Attenuated hindpaw mechanical withdrawal thresholds of spared nerve injury (SNI) mice on postoperative day (POD) 7 did not correlate with reduced Catwalk hindpaw pressure on POD 11 (n = 19, r(17) = −0.0077, p = 0.9750). Each data point represents one individual mouse. Correlation analyses were performed to calculate the Pearson correlation coefficient (r).

When changes in voluntary behavior were observed after inflammation or nerve injury, we performed correlation analyses to test whether the degree of change in voluntary behavior correlated to the degree of change in the mechanical withdrawal threshold for a given animal. Following bilateral CFA, attenuated hindpaw mechanical withdrawal thresholds on PID 0 were not significantly correlated with decreased voluntary wheel running on PID 1 (Fig. 4A, r(10) = −0.349, p = 0.267), as has been reported previously in rats (Grace et al., 2014). It has been suggested that hindpaw pressure measured via the Catwalk gait analysis system provides an objective readout of mechanical hypersensitivity. For instance, in some cases, a correlation has been reported between mechanical hypersensitivity and decreased hindpaw pressure following inflammation or nerve injury in rodents (Gabriel et al., 2007, Mogil et al., 2010, Vrinten and Hamers, 2003). However, we found no correlation between CFA-induced mechanical hypersensitivity on PID 0 and reduced Catwalk hindpaw pressure on PID 1 (Fig. 4B, r(21) = 0.248, p = 0.255). Likewise, following SNI, attenuated hindpaw withdrawal thresholds on POD 7 did not correlate with decreased Catwalk hindpaw pressure measured on POD 11 (Fig. 4C, r(17) = −0.0077, p = 0.9750). Taken together, our results demonstrate that neither inflammation- nor nerve injury-induced changes in voluntary behavior correlate to mechanical hypersensitivity. Thus, while each endpoint requires mice to ambulate on the injured hindpaw(s), voluntary wheel running and hindpaw pressure are not simply alternative measures of mechanical hypersensitivity.

### SNI-induced mechanical hypersensitivity, but not reduced hindpaw pressure, was reversed by an analgesic

**Fig. 5:**
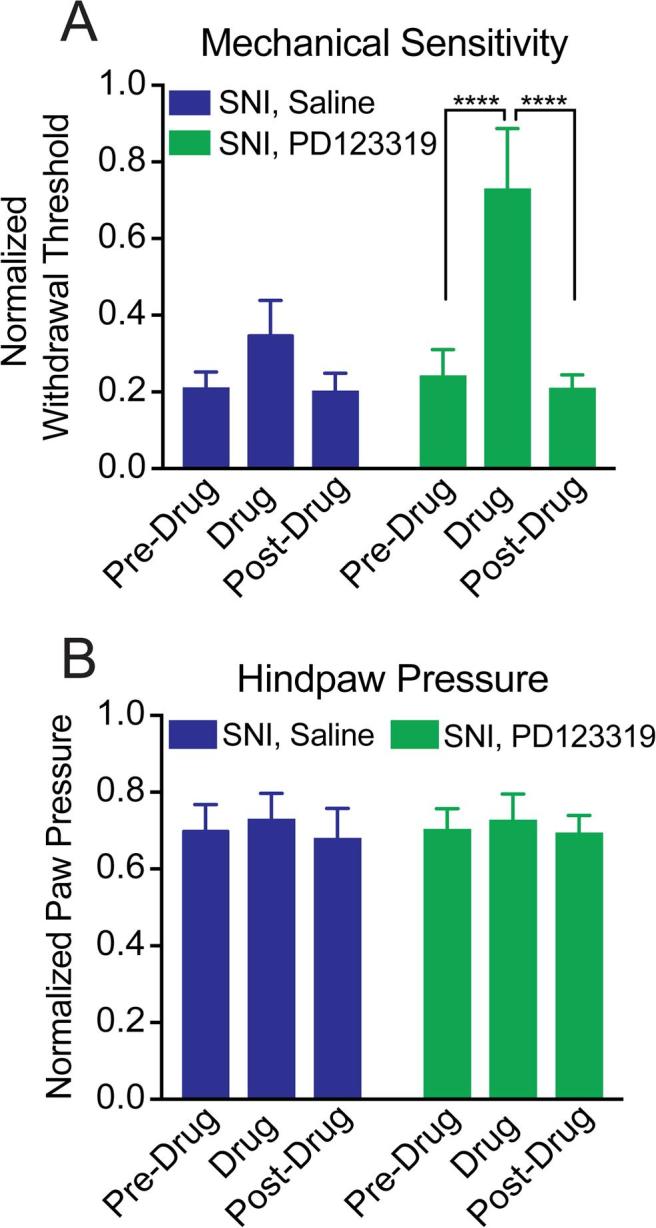
The analgesic PD123319 reversed SNI-induced mechanical hypersensitivity, but not SNI-induced decreases in Catwalk hindpaw pressure. (A). Systemic administration (10 mg/kg, i.p.) of the angiotensin II type 2 receptor antagonist PD123319, but not saline, significantly increased mechanical withdrawal thresholds of spared nerve injury (SNI) mice on postoperative days (POD) 12–15 or 26–30. Data from these timepoints were pooled together. (n = 11–12/group, two-way RM ANOVA, Sidak Correction: F(2,42) = 3.816, p = 0.03 for drug group × test session interaction). Mechanical withdrawal thresholds of SNI, PD123319 were significantly greater than SNI, saline mice following drug administration (Post-hoc Student’s t-Test: t = 3.018, p = 0.0110). Within the SNI, PD123319 group, drug administration significantly increased mechanical withdrawal thresholds compared to pre-drug and post-drug sessions. (Post-hoc Student’s t-Tests: for pre-drug compared to drug, t = 4.672, ^****^p < 0.0001; for post-drug compared to drug, t = 4.984, ^****^p < 0.0001). (B) Compared to pre-drug and post-drug sessions, or administration of saline, PD123319 did not change Catwalk hindpaw pressure of SNI mice on POD 9-12 (n = 5–8/group). Data are presented as mean ± SEM.

Lastly, we tested if the angiotensin II type 2 receptor antagonist PD123319 could reverse SNI-induced gait deficits to determine whether these were indeed pain-related changes. We were particularly interested in the effects of PD123319 on SNI-induced reductions in hindpaw pressure, which has been recommended as an objective measure of mechanical hypersensitivity (Coulthard et al., 2002, Gabriel et al., 2007, Pitzer et al., 2016, Vrinten and Hamers, 2003). Administration of PD123319 (10 mg/kg, i.p.), but not saline, significantly increased mechanical withdrawal thresholds of SNI mice (Fig. 5A, two-way RM ANOVA, Sidak correction: F(2,42) = 3.816, p = 0.03 for drug group x test session interaction). This interaction was driven by significantly increased mechanical withdrawal thresholds of SNI mice treated with PD123319 compared to those treated with saline on the day of drug administration (Post-hoc Student’s t-Test: t = 3.018, p = 0.0110), and significantly increased mechanical withdrawal thresholds within the PD123319 group on the drug session compared to pre-drug and post-drug sessions (Post-hoc Student’s t-Tests: for pre-drug compared to drug, t = 4.672, p < 0.0001; for post-drug compared to drug, t = 4.984, p < 0.0001). In contrast, compared to saline, administration of PD123319 did not significantly alter SNI-induced decreases in Catwalk hindpaw pressure, and no differences were observed within the PD123319 group across testing sessions (Fig. 5B). SNI-induced changes in hindpaw contact area, stance phase, and swing phase were similarly unaffected by PD123319 (data not shown). Furthermore, compared to pre-drug and post-drug sessions, PD123319 did not alter mechanical withdrawal thresholds or hindpaw pressure of the contralateral, uninjured hindpaw of SNI mice (data not shown). In summary, PD123319 significantly reversed SNI-induced mechanical hypersensitivity, but had no effect on SNI-induced reductions in Catwalk hindpaw pressure. Our findings contribute to the growing literature from both rats and mice demonstrating that nerve-injury induced gait deficits are not reversed by analgesics (Mogil et al., 2010, Piesla et al., 2009).

Although we also observed CFA-induced decreases in voluntary wheel running and Catwalk hindpaw pressure, we did not test whether these changes in voluntary behavior could be reversed by known analgesics. Cobos et al. have thoroughly demonstrated that non-steroidal anti-inflammatory drugs and morphine prevent inflammation-induced suppression of voluntary wheel running (Cobos et al., 2012). While to our knowledge prevention or reversal of gait deficits resulting from intraplantar CFA have not yet been demonstrated, we observed a relatively small decrease in hindpaw pressure in CFA mice (8.6% decrease from baseline, Fig. 1G), which provides little dynamic range for reversal by an analgesic.

## Discussion

In response to the widely critiqued translational gap between preclinical and clinical measures of pain (Barrot, 2012, Clark, 2016, Cobos and Portillo-salido, 2013, Mao, 2012, Mogil, 2009, Mogil and Crager, 2004, Rice et al., 2009, Sapunar and Puljak, 2009, Tappe-Theodor and Kuner, 2014, Vierck et al., 2008), we evaluated whether voluntary behaviors are interrupted in rodent models of persistent pain. Specifically, we tested for inflammation- and nerve injury-induced changes in voluntary wheel running, locomotion, gait, social interaction, and general anxiety-like behavior. There is currently conflicting evidence regarding the relevance of these endpoints as pain-related behaviors. However, in order to establish a new, reliable rodent pain-related behavior, it must be reversible by existing analgesics and validated by multiple independent investigators. In the present study, we demonstrate that inflammation and nerve injury minimally interfere with wheel running, locomotion, gait, social interaction, and anxiety-like behaviors in mice. Although significant nerve injury-induced gait deficits were observed, they were not reversed by the analgesic PD123319. Thus, we conclude that these voluntary behaviors are not reliable pain-related readouts across rodent injury models.

### CFA- and SNI-induced changes in physical activity are transient, if present

We tested whether voluntary measures of physical activity and mobility – wheel running, open field locomotion, and gait – are impaired in mice following CFA or SNI. We found that bilateral CFA reduced each measure of physical activity and mobility for up to 3 days post injection, suggesting that CFA transiently suppresses global physical activity in mice. These findings are in close alignment with similar studies of CFA-induced decreases in voluntary wheel running (Cobos et al., 2012, Grace et al., 2014, Kandasamy et al., 2016, Pitzer et al., 2016). In contrast, unilateral SNI impaired gait (see below), but otherwise did not interfere with physical activity or mobility. Previous studies support our finding that open field locomotion is unaffected by nerve injury (Cho et al., 2013, Mogil et al., 2010, Urban et al., 2011, Yalcin et al., 2011, Zhou et al., 2015). However, our data demonstrating that nerve injury does not suppress wheel running raises questions.

A distinguishing feature of our wheel running paradigm is that mice were provided acute (2 h), rather than homecage (24 h) wheel access, which could have influenced our null observation. However, existing studies in which rodents were provided homecage wheel access provide conflicting evidence regarding voluntary wheel running as a pain-related behavioral endpoint following nerve injury. Pitzer et al. report that SNI attenuated homecage wheel running distances in mice, and differences between sham and SNI mice were only apparent during the dark cycle (Pitzer et al., 2016). However, Grace et al. observed no difference in homecage running distances between sham and CCI rats (Grace et al., 2016), and our previous work similarly found no difference between running distances of naïve and SNI mice that were given acute access for 2 or 12 h/dark cycle (Sheahan et al., 2015).

Another noteworthy feature of our nerve injury studies is that SNI was unilateral. We and others have found that bilateral CFA, but not unilateral CFA, suppresses voluntary wheel running (Cobos et al., 2012). These results suggest that rodents may be better able to adapt to unilateral hindpaw injury compared to bilateral injury. Whether the nerve injury model, injury laterality, testing time of day, or extent of wheel access prior to and/or following induction of pain underlie these discrepancies requires further investigation. Collectively, our present and previous data support that voluntary wheel running, and more broadly physical activity, is reliably suppressed for a short time post-injury by inflammation, but not by nerve injury.

Whether gait alterations represent pain-related/avoidance behaviors across rodent injury models is unclear (Chiang et al., 2014, Coulthard et al., 2002, Mogil et al., 2010, Parvathy and Masocha, 2013, Piesla et al., 2009, Pitzer et al., 2016, Pitzer et al., 2016, Robinson et al., 2012, Urban et al., 2011). We found that both CFA and SNI reduced hindpaw pressure of the affected limb(s). In addition, SNI altered dynamic gait parameters including stance and swing phase, while CFA did not. The use of bilateral CFA versus unilateral SNI may underlie the apparent differences between the effects of inflammation and nerve injury on dynamic gait parameters within our study. In fact, previous rodent gait analysis studies of both unilateral inflammatory and nerve injury pain models show significant changes in stance and swing phases (Chiang et al., 2014, Coulthard et al., 2002, Mogil et al., 2010, Piesla et al., 2009, Pitzer et al., 2016, Pitzer et al., 2016).

Interestingly, we found that compared to the duration of mechanical hypersensitivity, gait alterations following CFA were transient, whereas gait changes following SNI persisted equally as long as mechanical hypersensitivity. As the SNI model involves ligating both sensory and motor axons, we hypothesized that SNI-induced gait changes were due in part to a motor deficit, and our data suggest that this is the case. First, like others (Urban et al., 2011), we found that SNI impaired gross motor coordination measured via the rotarod test. Similarly, our previous results show that SNI mice were impaired in the inverted screen test and had significantly atrophied gastrocnemius muscles (Sheahan et al., 2015), further demonstrating the impact of SNI on motor axons. Second, although nerve injury-induced gait changes have been proposed to reflect pain-related behavior (Pitzer et al., 2016), to our knowledge, only one previous study has evaluated the effect of analgesics on Catwalk gait deficits in mice (Mogil et al., 2010). We show that SNI-induced gait changes were not reversed by the analgesic PD123319. Together, these findings indicate that nerve injury induced-changes in gait are not driven by pain; rather, they are likely the product of a motor deficit. In contrast, it is likely that pain underlies inflammation-induced changes in gait, which have been successfully reversed by analgesics (Adams et al., 2016, Piesla et al., 2009, Robinson et al., 2012). These observations emphasize that demonstrating reversal via known analgesics is a crucial step in establishing new pain-related behaviors.

Clinically, it is not uncommon for analgesics to reverse hypersensitivity, yet fail to improve other aspects of chronic pain (Dworkin et al., 2005). Indeed, assays used in the present study likely generate differing nociceptive inputs: the von Frey test of mechanical hypersensitivity entails focal hindpaw mechanical stimulation, whereas voluntary behavior assays such as gait analysis or wheel running reflect nociceptive inputs integrated from the entire hindpaw. Thus, it is possible our observation that PD123319 reverses SNI-mechanical hypersensitivity, yet fails to reverse SNI-induced gait deficits, reflects differences in analgesic efficacy of PD123319 on differing nociceptive stimulus inputs. However, this possibility is contradicted by the finding that PD123319 successfully reverses SNI-induced changes in voluntary behavior in assays that similarly vary in nociceptive inputs, including the warm/cool plate avoidance system as well as the mechanical avoidance assay (Shepherd et al., 2017).

### Neither CFA nor SNI alter social interactions

There is growing evidence from clinical and preclinical studies that chronic pain both influences and is influenced by social interactions (Langford et al., 2010, Martin et al., 2015, Martin et al., 2014, Mogil, 2015, Pitzer et al., 2016, Pitzer et al., 2016, Smith et al., 2016). For instance, patients report that pain substantially interferes with social activities and relationships (Jensen et al., 2007, Nicholson and Verma, 2004, Wallace et al., 2014). Like humans, mice partake in complex social interactions (Silverman et al., 2010, Terranova and Laviola, 2005). The social approach assay utilized here encompasses a combination of perhaps conflicting motivations including social investigation, play, offensive aggression, and/or perception of the stimulus mouse as a stressor (Brodkin et al., 2004). We found no effect of CFA or SNI on C57BL/6J social interactions on PID 2 or POD 8/14, respectively. Previous studies have similarly shown that social interactions are unchanged in mice following inflammation (Liu et al., 2015, Urban et al., 2011), and reduced acutely (POD 5) after nerve injury, if at all (Urban et al., 2011, Zhou et al., 2015). These data suggest that changes in social interaction do not reliably manifest in common mouse models of persistent pain. Notably, C57BL/6J mice display high sociability compared to other mouse strains (Brodkin et al., 2004, Sankoorikal et al., 2006), which may mask the effects of injury on social behavior. Thus, the impact of inflammation and nerve injury on social interaction across strains requires further investigation.

### Neither CFA nor SNI induce anxiety-like behavior

There is an increased prevalence of anxiety in chronic pain patients (Gureje, 2008, Jensen et al., 2007). To test whether persistent pain similarly induces anxiety-like behavior in mice, we utilized the open field test and found that neither CFA nor SNI elicited anxiety-like behavior during ongoing mechanical hypersensitivity. Previous studies using the open field test have also reported a lack of anxiety-like behavior in mice up to 4 weeks after inflammation or nerve injury (Liu et al., 2015, Urban et al., 2011). However, anxiety-like behavior has been detected in rodents using the elevated plus maze after both inflammation and nerve injury, and is reversed by anxiolytics and analgesics (Dimitrov et al., 2014, Liu et al., 2015, Narita et al., 2006, Parent et al., 2012, Refsgaard et al., 2016, Roeska et al., 2009). Therefore, it is possible that CFA and SNI mice would have exhibited anxiety-like behavior if we had used additional measures of anxiety-like behavior.

### Hypersensitivity and suppressed voluntary behaviors represent different aspects of pain

We demonstrated that changes in mechanical withdrawal thresholds and voluntary behaviors represent distinct components of inflammation and nerve injury. We found no correlation between CFA- and/or SNI-induced mechanical hypersensitivity and decreased Catwalk hindpaw pressure or voluntary wheel running. Similarly, studies of other rodent voluntary behaviors such as burrowing and sleep cycle report no correlation with hypersensitivity (Lau et al., 2013, Monassi et al., 2003). These results support the idea that changes in paw-withdrawal reflexes and voluntary behavior are driven by different pathophysiologies (Mogil and Crager, 2004, Urban et al., 2011). For instance, compared to mechanical withdrawal reflexes, voluntary wheel running is a complex behavior that engages reward circuitry (Brene et al., 2007, Novak et al., 2012). In turn, disruption of this behavior by pain likely reflects a combination of somatosensory, affective, and motivational changes.

### Changes in voluntary behavior are not characteristic of persistent pain in mice

Improving the translatability of basic research findings is a priority to the field. In turn, considerable efforts have been directed towards identifying measures of spontaneous pain in rodent models. While impairment of voluntary behaviors and quality of life measures have successfully been reversed by analgesics in rodent models of acute pain (Miller et al., 2011, Stevenson et al., 2006, Stevenson et al., 2009), the present study and others demonstrate that these endpoints are less informative in common rodent models of persistent pain (Mogil et al., 2010, Urban et al., 2011). In most cases, there are either no or only modest, short-lived changes in voluntary behavior, which limit the ability to study the time course of pain pathologies and the efficacy of novel analgesics. These observations raise two important possibilities. Foremost, spontaneous pain may be either short-lived or well-masked in mice because as prey animals, it is evolutionarily disadvantageous for mice to display signs of injury or weakness. Second, while mice certainly display sensitization after injury, it is possible that mice do not experience pain as a complex sensory and emotional state as humans do. Both of these possibilities could represent a formidable challenge of using mice to model the negative impact of chronic pain on the quality of life of humans.

Despite these obstacles, there are a variety of promising tools to bridge the translational gap between rodent and human pain research. For instance, operant and classical conditioning assays such as the mechanical conflict system and conditioned place preference/aversion possess predictive validity as measures of motivational aspects of pain (Harte et al., 2016, Navratilova et al., 2013). Further, agnostic approaches to analyzing rodent body language and behavioral phenotypes may reveal novel endpoints that access the presence of ongoing sensitization in rodents without anthropomorphizing (McCall et al., 2017, Wiltschko et al., 2015). Thus, although we found minimal effects of inflammation and nerve injury on mouse physical activity, social interaction, or anxiety-like behavior, using operant assays or agonistic approaches in conjunction with traditional measures of nociceptive thresholds may aid in increasing the translation of preclinical findings.

## Disclosures

The authors have no conflicts of interest to declare.

## Funding

This work was supported by the National Institute of Neurological Disorders and Stroke grants R01NS069898 to DPM and R01NS042595 to RWG.
